# Identification of congenital rubella syndrome in Sudan

**DOI:** 10.1186/1471-2334-14-305

**Published:** 2014-06-04

**Authors:** Omer Adam, Ahmed KM Ali, Judith M Hübschen, Claude P Muller

**Affiliations:** 1 Department of Medical Biotechnology, Commission for Biotechnology and Genetic Engineering, The National Centre for Research, P. O. Box 2404, Khartoum, Sudan; 2 Institute of Immunology, Centre de Recherche Public de la Santé/Laboratoire National de Santé, 20A Rue Auguste Lumière, L-1950 Luxembourg, Luxembourg

**Keywords:** Congenital rubella syndrome, Eye defects, Oral fluid, Dried blood spot, Sudan

## Abstract

**Background:**

Epidemiological data about congenital rubella syndrome (CRS) are scarce and rubella vaccine is not yet included in the childhood immunization schedule in Sudan. This study aimed to identify and describe CRS cases among Sudanese infants with congenital eye or heart defects.

**Methods:**

Between February and September 2010, paired oral fluid and dried blood spot samples were collected from 98 infants aged up to 12 months. These infants were enrolled during their visits to five hospitals in Khartoum, Sudan. Clinical samples were screened for rubella IgM and for ≥ 6 months old infants also for IgG antibodies by ELISA. The oral fluid of IgM and/or IgG positive patients was tested for rubella RNA by reverse transcriptase PCR.

**Results:**

Our findings revealed that two children (2.0%) were IgM positive and another five children (5.1%) were positive for IgG antibodies. None of the five infants of which enough oral fluid was available for RNA investigation was PCR positive.

**Conclusions:**

This study documented the presence of CRS in Sudan and highlighted the importance of rubella vaccine introduction for preventing future CRS cases in the country.

## Background

Rubella (German measles) is a common febrile rash illness caused by rubella virus. Rubella occurs mostly during childhood usually as a mild or even asymptomatic infection [1]. However, infection during the first trimester of pregnancy can lead to a spectrum of birth defects known as congenital rubella syndrome (CRS) including congenital eye defects, deafness, congenital heart diseases and mental retardation [2]. CRS can be prevented through rubella vaccination available since 1969 [3]. The incidence of CRS has been reduced in many developed countries by effective vaccination programs [4]. Effective rubella vaccination programs as well as high-quality surveillance of rash/fever diseases have been implemented in the Americas and resulted in rubella and CRS elimination in those countries since 2010 [5,6]. However, rubella vaccination has not yet been introduced in many developing countries [7]. The burden of CRS in these countries is underestimated and few reports documenting the incidence of CRS are available. In 2009, only 165 CRS cases were reported worldwide with the majority being from the World Health Organization (WHO) African and Eastern Mediterranean regions [8].

In Sudan, national surveillance for measles and rubella was established in 2006. However, neither routine CRS surveillance nor rubella vaccination is available and data on CRS are inadequate. We documented the occurrence of CRS in Sudan for the first time in 2010 [9] and reports about rubella seroprevalence among pregnant women are available from some Sudanese states including Khartoum State [10] and West Sudan [11]. The present study aimed to identify CRS cases among Sudanese infants presented at different hospitals in Khartoum to obtain more information about the CRS situation in Sudan. These data may help public health authorities to design appropriate CRS prevention strategies.

## Methods

### Study settings

This cross-sectional study was conducted between February and September 2010 to identify CRS cases among infants presented to five hospitals in Khartoum, Sudan. Khartoum with an estimated 5 million residents in 2008 [12] is considered to be a major centre for medical facilities, also attracting patients from across the country. The hospitals selected for the present study (two ophthalmology hospitals, two paediatrics hospitals and a paediatrics echocardiography unit) are major specialised hospitals that provide paediatric services for a large number of Sudanese children.

### Study participants

The initial selection of the study subjects was based on the WHO case definitions [13] and both suspected and clinically-confirmed CRS cases were included (Table 1). Physicians were provided with the study inclusion/exclusion criteria before the start of the study. A total of 98 infants aged up to 12 months who matched these case definitions were recruited during the study period and their samples were tested for laboratory confirmation. The clinical examination of these cases was done by qualified physicians according to the specialty of the hospital. As hearing loss was not evaluated in this study, we included infants who presented either with congenital eye defects, heart defects or both. Children aged more than 12 months or presenting with congenital defects not compatible with the CRS case definition were excluded. At the ophthalmology hospitals and the echocardiography unit all infants matching the inclusion criteria and presenting during the 7-months study period were included. For the paediatrics hospitals the research team was called upon by hospital staff when patients matching the inclusion criteria were presented. Clinical symptoms compatible with CRS detected during medical examination, extracted from the infants’ medical records or described by their parents were recorded.

**Table 1:** WHO case definitions for congenital rubella syndrome (CRS) and congenital rubella infection (CRI) *

**Case definition**	**Description**
**Suspected CRS case**	Any infant less than one year of age in whom a health worker suspects CRS. A health worker should suspect CRS when the infant presents with heart disease, and/or suspicion of deafness, and/or one or more of the following eye signs: white pupil (cataract); diminished vision; pendular movement of the eyes (nystagmus); squint; smaller eye ball (micropthalmos); larger eye ball (congenital glaucoma). A health worker should suspect CRS where there is a maternal history of suspected or confirmed rubella during pregnancy.
**Clinically-confirmed CRS case**	A clinically-confirmed case is one in which a qualified physician detects two of the complications in section (a) OR one from group (a) and one from group (b):
	(a) Cataract(s) and/or congenital glaucoma; congenital heart disease; loss of hearing; pigmentary retinopathy.
	(b) Purpura; splenomegaly; microcephaly; mental retardation; meningoencephalitis; radiolucent bone disease; jaundice with onset within 24 hours after birth.
**Laboratory-confirmed CRS case**	A laboratory-confirmed CRS case is an infant with a positive blood test for rubella IgM who has clinically-confirmed CRS.
**Congenital rubella infection (CRI)**	An infant with a positive blood test for rubella IgM who does not have clinically-confirmed CRS is classified as having congenital rubella infection (CRI).

*Source: WHO (1999) [13].

### Ethics

This study was reviewed and approved by the Health Research Ethics Committee, Ministry of Health, Sudan. Parents of infants that met the CRS clinical case definition were informed about the study and consented prior to study enrolment.

### Sample and data collection

Paired samples of oral fluid (OF) and dried blood spots (DBS) were collected from each participant. The OF samples were collected with the ORACOL collection device (Malvern Medical Developments, Worcester, UK) and prepared as previously described [14]. The DBS samples were collected on Whatman® No. 903 filter paper and eluted as recommended in the manual for the laboratory diagnosis of measles and rubella virus infection [15]. A questionnaire including age, gender and place of residence of the infant, clinical signs, age of the mother and maternal history of rash and rubella vaccination was completed for each participating infant. While the clinical data were recorded by the physicians, other data were gathered by the research team from the mother. Access to the completed questionnaires was restricted to members of the research team only.

### Laboratory testing

The laboratory confirmation of CRS cases was based on the detection of rubella IgM antibodies. Both OF and DBS samples of all 98 participating infants were investigated for specific IgM antibodies using the Microimmune Rubella IgM capture EIA kit (Microimmune Limited, UK) and the Anti-rubella Virus IgM ELISA kit (Siemens, Germany), respectively. According to the manufacturers these tests have a specificity and sensitivity of at least 96.9%. The DBS samples of the 49 infants aged ≥ 6 months were also screened for rubella IgG antibodies using the Anti-rubella Virus IgG ELISA kit (Siemens, Germany; specificity 98.5% and sensitivity 100% according to the manufacturer) since the persistence of rubella IgG antibodies in infants beyond that age may be suggestive of CRS [16-18]. From five of the children who were IgM and/or IgG positive, enough OF was left for RNA extraction and reverse transcription PCR using previously published primers [19,20] and to attempt virus isolation [15].

### Data analysis

The data recorded on the questionnaires were analyzed using SPSS software version 12.0 (SPSS Inc. USA).

## Results

The age of the enrolled infants ranged between 1 day to 12 months (median age 5.5 months) and 50.0% of them were < 6 months old. 65.3% were males and 34.7% were females. Most of the infants were from Khartoum (38.8%) and Gazeera (22.4%) states and the remaining children (38.8%) were from various other Sudanese states. The majority of the enrolled infants (89.8%) were presented to the ophthalmology hospitals and only 8.2% and 2.0% were seen in the echocardiography unit and the paediatrics hospitals, respectively. At presentation, 24.5% of the infants were classified as clinically-confirmed CRS cases (median age 4 months; 13 cases had congenital eye defects, 4 infants had congenital heart defects and 7 children had both eye and heart defects) and 75.5% as suspected CRS cases (median age 6 months; 74 cases with congenital eye defects, none had congenital heart defects) according to the WHO case definitions [13]. Congenital cataract was the most frequent clinical presentation (48.0%) among the investigated infants, followed by congenital glaucoma (33.7%) and congenital heart defects (11.2%). The analysis of maternal history revealed that the median age of the mothers was 25 years (range 14 to 45 years), none of them was vaccinated against rubella and only 5 mothers (5.1%) had febrile rashes during their pregnancy.

Our results showed that two infants aged 3 and 9 months were positive for rubella IgM in both OF and DBS samples (case 1 and case 2). Case 1 presented with congenital glaucoma only and was thus classified as a case of congenital rubella infection, while case 2 had congenital cataract, pigmentary retinopathy and micropthalmia and was classified as a laboratory-confirmed case (Table 2). Five infants aged ≥ 6 months were considered as potential CRS cases as they were positive for rubella IgG antibodies (case 3 till case 7). Two of them were clinically-confirmed CRS cases (case 3 and case 4), while the other three were suspected cases (case 5, 6 and 7) (Table 2). For one of the clinically-confirmed cases the mother reported that she had febrile rash illness during pregnancy (case 3). None of the five tested IgM and/or IgG positive cases was PCR or virus culture positive.

**Table 2:** Characteristics, clinical signs, case classifications and rubella antibodies among seven confirmed or potential CRS cases

**Cases**	**Age (months)**	**Clinical signs**	**WHO definitions for CRS cases**	**IgM (OF)**	**IgM (DBS)**	**IgG (DBS)**	**History of Maternal rash**
Case 1	3	Glaucoma	Congenital rubella infection	**+**	**+**	NT	No
Case 2	9	Cataract, pigmentary retinopathy, microphthalmia	Laboratory-confirmed CRS	**+**	**+**	**+**	No
Case 3	6	Cataract, CHD (VSD), splenomegaly, jaundice	Clinically-confirmed CRS^#^	**–**	**–**	**+**	Yes
Case 4	6	Glaucoma, CHD (PDA, ASD)	Clinically-confirmed CRS^#^	**–**	±	**+**	No
Case 5	6	Pigmentary retinopathy	Suspected CRS^#^	**–**	**–**	**+**	No
Case 6	6	Cataract	Suspected CRS^#^	**–**	**–**	**+**	No
Case 7	12	Micropthalmia, purpura	Suspected CRS^#^	**–**	**–**	**+**	No

ASD = atreial septal defect; CHD = congenital heart disease; NT = Not Tested; PDA = Patent ductus arteriosus; VSD = Ventricular septal defect; # = potential CRS case.

## Discussion

In many developing countries, the burden of CRS is under-estimated [2]. Also Sudan lacks robust information about the burden of CRS, although this information is important for the decision to introduce rubella-containing vaccine in the national immunization program. Our results showed that of 98 infants with symptoms compatible with CRS, two had specific rubella IgM antibodies, one of which was classified as a laboratory-confirmed case and the other as a congenital rubella infection case. Another five infants ≥ 6 months were positive for rubella IgG antibodies and considered potential CRS cases because routine rubella vaccination is not yet practiced in Sudan and postnatal rubella infections seem to be uncommon among infants less than one year of age [16]. In addition, none of the mothers was vaccinated and all infants had clinical signs compatible with CRS. However, it is possible that maternal antibodies acquired after rubella infection still persist in 6–12 months old infants [21]. This may be especially the case in the four 6 months old children in our study (case 3 till case 6), while it is less likely for the 12 months old child (case 7).

The remaining 91 subjects, including 21 clinically confirmed cases, were negative for both rubella IgM and IgG antibodies. A potential explanation is that the observed defects were caused by other pathogens involved in congenital disorders such as *Toxoplasma gondii*, cytomegalovirus or herpes simplex virus [22]. This hypothesis is supported by rubella surveillance data from Sudan for 2009 and 2010, which showed that only about 300 rubella cases were identified per year (343 cases in 2009 and 302 in 2010) [23]. Thus, it would be interesting to investigate samples from suspected CRS cases that cannot be confirmed in the laboratory for other possible etiologies to clarify their role in congenital disorders detected in Sudan.

The current report again documented the presence of CRS in Sudan. Previously, we identified CRS in 11 Sudanese infants using ELISA, reverse transcriptase-PCR and rubella virus isolation [9]. Another recent study identified seven cases of congenital rubella infection among 92 newborns in Khartoum based on ELISA testing of cord blood [24]. As a high rubella seronegativity rate of 34.7% was recently detected among pregnant women in the Western region of Sudan [11], new CRS cases are likely to occur. In contrast, a much lower rubella seronegativity rate of 4.9% was observed in Khartoum State [10], but CRS may still occur even when susceptibility levels are below 10% [13].

In this study, we used OF and DBS samples to avoid the difficulty of blood sampling from infants. Both samples have been described as alternatives to serum for rubella surveillance, as they exhibit equivalent sensitivity and specificity [24]. Here, we observed a high concordance of 95.9% (94/98) between the rubella IgM results in the paired OF and DBS samples, and only 4 (4.1%) of the sample pairs had discrepant results (2 patients negative in the OF and equivocal in the DBS and 2 times vice versa). Initially the OF samples of two other patients tested positive while the corresponding DBS were negative, but upon repetition of the OF testing the positive result was not confirmed. The discrepancies between OF and DBS testing in this study, as well as the low number of laboratory-confirmed CRS cases could be related to inappropriate collection of these samples [25]. Given the advantages of these alternative sampling techniques and the high concordance between the two specimen types, alternative samples should nevertheless be considered for future CRS research in Sudan.

With the current study, we further emphasized CRS as a public health burden in Sudan. However, this study was subject to some limitations including the short participant recruitment period, the limited number of participating hospitals and the lack of clinical examination of hearing deficits, another common symptom of CRS. Despite these limitations, our results together with the findings of other recent studies from Sudan highlight the need of introducing rubella vaccination in Sudan. Rubella vaccination has been shown to be cost-effective [7], while the treatment of CRS even in poor countries is very costly. The WHO recommends that all countries that have not yet introduced rubella vaccine should consider its inclusion in their national immunization programme [3]. As Sudan has achieved a measles vaccine coverage of >80% during the past few years, the country meets the WHO criteria for introducing rubella vaccine [7]. Strengthening of the currently existing measles and rubella surveillance system and eventually its extension to rash/fever disease surveillance would further support disease control efforts.

## Conclusions

This study again documented the presence of CRS in Sudan. It also highlighted the importance of rubella vaccination for the interruption of rubella virus transmission to prevent future CRS cases in Sudan.

## Abbreviations

CRS: Congenital rubella syndrome; EIA: Enzyme Immunoassay; ELISA: Enzyme-linked immunosorbent assay; DBS: Dried blood spot; IgG: Immunoglobulin G; IgM: Immunoglobulin M; OF: Oral fluid; PCR: Polymerase chain reaction; SPSS: Statistical package for the social sciences; WHO: World Health Organization.

## Competing interests

The authors declare that they have no competing interests.

## Authors’ contributions

OA designed the study, collected the samples, did the experimental work and statistical analysis, and wrote the manuscript. AKMA contributed to drafting the manuscript. JMH was involved in the conception of the study, the interpretation of the results and the writing of the manuscript. CPM revised the manuscript. All authors read and approved the final manuscript.

## Pre-publication history

The pre-publication history for this paper can be accessed here:

http://www.biomedcentral.com/1471-2334/14/305/prepub
